# Correlation between arterial blood pressures and regional cerebral oxygen saturation in preterm neonates during postnatal transition-an observational study

**DOI:** 10.3389/fped.2022.952703

**Published:** 2022-09-06

**Authors:** Daniel Pfurtscheller, Christina H. Wolfsberger, Nina Höller, Bernhard Schwaberger, Lukas Mileder, Nariae Baik-Schneditz, Berndt Urlesberger, Gerhard Pichler

**Affiliations:** ^1^Research Unit for Neonatal Micro- and Macrocirculation, Department of Pediatrics and Adolescent Medicine, Medical University of Graz, Graz, Austria; ^2^Division of Neonatology, Department of Pediatrics and Adolescent Medicine, Medical University of Graz, Graz, Austria

**Keywords:** blood pressure (BP), neonate-age, transition, cerebral oxygenation (O2Hb), cerebral autoregulation

## Abstract

**Objective:**

To assess whether blood pressure (systolic (SABP), diastolic (DABP), and mean arterial blood pressure (MABP) and cerebral-regional-oxygen-saturation (crSO2) and cerebral-fractional-tissue-oxygen-extraction (cFTOE) are associated after immediate fetal-to-neonatal transition in preterm neonates with and without respiratory support.

**Study design:**

*Post-hoc* analyses of secondary outcome parameters of prospective observational studies were performed. We included moderate and late preterm neonates with and without respiratory support with cerebral NIRS monitoring (INVOS 5100c) and an oscillometric blood pressure measurement at minute 15 after birth. Heart rate (HR) and arterial oxygen saturation (SpO2) were monitored routinely. Blood pressure values were correlated with crSO2 and cFTOE.

**Results:**

47 preterm neonates with NIRS measurements and blood pressure measurement during immediate transition after birth were included. Twenty-five preterm neonates (gestational age: 34.4±1.6 weeks) received respiratory support. In these neonates crSO2 correlated significantly positively with systolic blood pressure (SABP; r = 0.46, *p* = 0.021), diastolic blood pressure (DABP; r = 0.51, *p* = 0.009) and, mean arterial pressure (MABP; r = 0.48, *p* = 0.015). cFTOE correlated significantly negatively with SABP (r = −0.44, *p* = 0.027), DABP (r = −0.49, *p* = 0.013) and mean MABP (r = −0.44, *p* = 0.029). Twenty-two preterm neonates (gestational age: 34.5 ± 1.5 weeks) did not receive respiratory support. In those neonates, neither crSO2 nor cFTOE correlated with blood pressure.

**Conclusion:**

In compromised moderate and late preterm neonates with respiratory support, both, crSO2 and cFTOE correlated with blood pressure. These findings suggest that passive pressure-dependent cerebral perfusion was present in preterm neonates with respiratory support, indicating an impaired cerebral autoregulation in those compromised preterm neonates.

## Introduction

Advances in neonatal medicine allow for increased survival rates of preterm neonates. However, the decrease in mortality is accompanied by an increased risk of neurodevelopmental deficits (1). The period of transition from fetal-to-neonatal life is associated with significant changes in oxygenation and systemic circulation, thus representing a vulnerable period for impairment of organ perfusion (2). Especially preterm neonates are at increased risk of phases of cerebral hypoxia and hyperoxia due to immaturity of cerebral autoregulation and, thus, potentially having an increased risk of cerebral impairment (3, 4).

Cerebral regional oxygen saturation (crSO2) depends on both, oxygen delivery and consumption. The oxygen delivery is dependent on the hemoglobin concentration of the blood, arterial oxygen saturation (SpO2) and cerebral perfusion (5, 6). The latter relies on the vascular resistance and cerebral perfusion pressure (CPP), which is defined by the relation between intracranial pressure and arterial blood pressure (7). Intact cerebral autoregulation enables constant cerebral blood flow (CBF) and is independent of the cerebral perfusion pressure, as long as the blood pressure is within the range of the autoregulatory plateau (8, 9). Above the autoregulatory plateau, the CBF increases, and below the autoregulatory plateau the CBF decreases in a pressure-passive manner (10, 11). Impaired autoregulation leads to a positive correlation between blood pressure and CBF as a result of a passive pressure-dependent cerebral perfusion. This passive pressure-dependent cerebral perfusion leads to deleterious CBF due to CPP variation.

crSO2 can be monitored continuously and non-invasively with near-infrared spectroscopy (NIRS) (12, 13). This technology uses the differences in absorbed light of the chromophores of oxygenated and deoxygenated hemoglobin. Cerebral fractional tissue oxygen extraction (cFTOE) can be calculated from crSO2 and SpO2.

Several studies have already investigated cerebral oxygenation and cardio-circulation (14–19). However, to the author's knowledge, only Baik et al. (20) compared NIRS and blood pressure parameters within the first 15 min after birth.

Thus, the aim of this study was to examine whether there is a potential correlation between blood pressure [systolic (SABP), diastolic (DABP), and mean arterial blood pressure (MABP)] with crSO2 and cFTOE after the immediate transition period at 15 min after birth in stable preterm neonates and those receiving respiratory support during the immediate transition period. We hypothesized that compromised preterm neonates who receive respiratory support have impaired cerebral autoregulation and, therefore, higher blood pressure values are associated with higher crSO2 and lower cFTOE values, whereas in stable preterm neonates cerebral autoregulation keeps crSO2 and cFTOE independent of blood pressure.

## Materials and methods

### Design

In the present observational study *post hoc* analyses of secondary outcome parameters of prospective observational studies (14, 21–23) conducted at the Division of Neonatology, Department of Pediatrics and Adolescent Medicine, at the Medical University of Graz, Austria, between July 2009 and September 2018 were performed. The Regional Ethics Committee approved the studies (EC numbers: 19–291 ex 07/08, 23–403 ex 10/11, and 27–465 ex 14/15). Written parental consent was obtained before birth for all neonates included in each study.

### Inclusion and exclusion criteria

Preterm neonates delivered by cesarean section, who received cerebral NIRS monitoring during the immediate transition period after birth and who had additional blood pressure measurements obtained at 15 min after birth, were included in the present *post hoc* analyses. Exclusion criteria were major congenital malformations and term gestational age.

### Monitoring

Demographics and antepartum medical history of neonates were collected from patient charts. All neonates measured were routinely transferred to the resuscitation table immediately after birth.

During the first 15 min after birth, SpO2 and the HR were routinely obtained by pulse oximetry, with the sensor being placed on the right hand or wrist (Intelli Vue MP 30 Monitor, Philips, Amsterdam, The Netherlands). crSO2 was measured by NIRS, which was performed with a cerebral oximeter monitor (INVOS 5100c, Medtronic, Minneapolis, Minnesota USA) using a neonatal sensor. The sensor was placed on the left frontoparietal head of the neonate and secured with an elastic bandage (Peha-haft, Harmann, Heidenheim, Germany) or a modified continuous positive airway pressure (CPAP) cap. cFTOE was calculated with the following equation: cFTOE = (SpO2–crSO2)/SpO2.

Arterial blood pressure was measured non-invasively in minute 15 after birth with a standard oscillometric blood pressure cuff on the right calf (Intelli Vue MP 30 Monitor, Philips, Amsterdam, The Netherlands).

All data were stored continuously in a polygraphic system (alpha trace digital MM, BEST Medical Systems, Vienna, Austria) for further analyses.

Included preterm neonates were then subsequently separated into two groups, depending on whether they received respiratory support (CPAP or positive pressure ventilation) or not during the first 15 min after birth.

### Statistical analysis

Demographic information, routine monitoring data, NIRS data and blood pressure measurements are presented as mean and standard deviation (SD) for normally distributed data or as median and interquartile range (IQR) for not normally distributed data.

For comparisons of baseline characteristics between preterm neonates with and without respiratory support for non-continuous variables the Chi-square test or Fisher's exact test were used, and for continuous variables Student's *t*-test or Mann-Whitney U test were applied.

Correlation analyses between blood pressure values (SABP, DABP and MABP) and in addition HR at minute 15 after birth and NIRS parameters (crSO2 and cFTOE) at minute 15 after birth were calculated for preterm neonates with and without respiratory support using Pearson's correlation for normally distributed data and Spearman's rank correlation for skewed distributions.

A *p-value* < 0.05 was considered statistically significant. These values were considered in an explorative sense. Therefore, no multiple testing corrections were performed. All statistical analyses were performed using IBM SPSS Statistics 26 (IBM Corporation, Armonk, NY, USA).

## Results

Between July 2009 and September 2018. 659 preterm and term neonates were included in the prospective observational studies. 511 term neonates were excluded. Out of the 148 remaining preterm neonates 47 preterm neonates were included with both NIRS and blood pressure measurements at minute 15 after birth. Indications for preterm birth were preterm labor (respiratory support *n* = 13 / no respiratory support *n* = 9), preterm premature rupture of membranes (*n* = 5 / *n* = 2), preeclampsia / HELLP syndrome / maternal hypertension (*n* = 2 / *n* = 3), intrauterine growth restriction (*n* = 2 / *n* = 5), and other reasons (*n* = 3 / *n* = 3).

**Figure 1:**
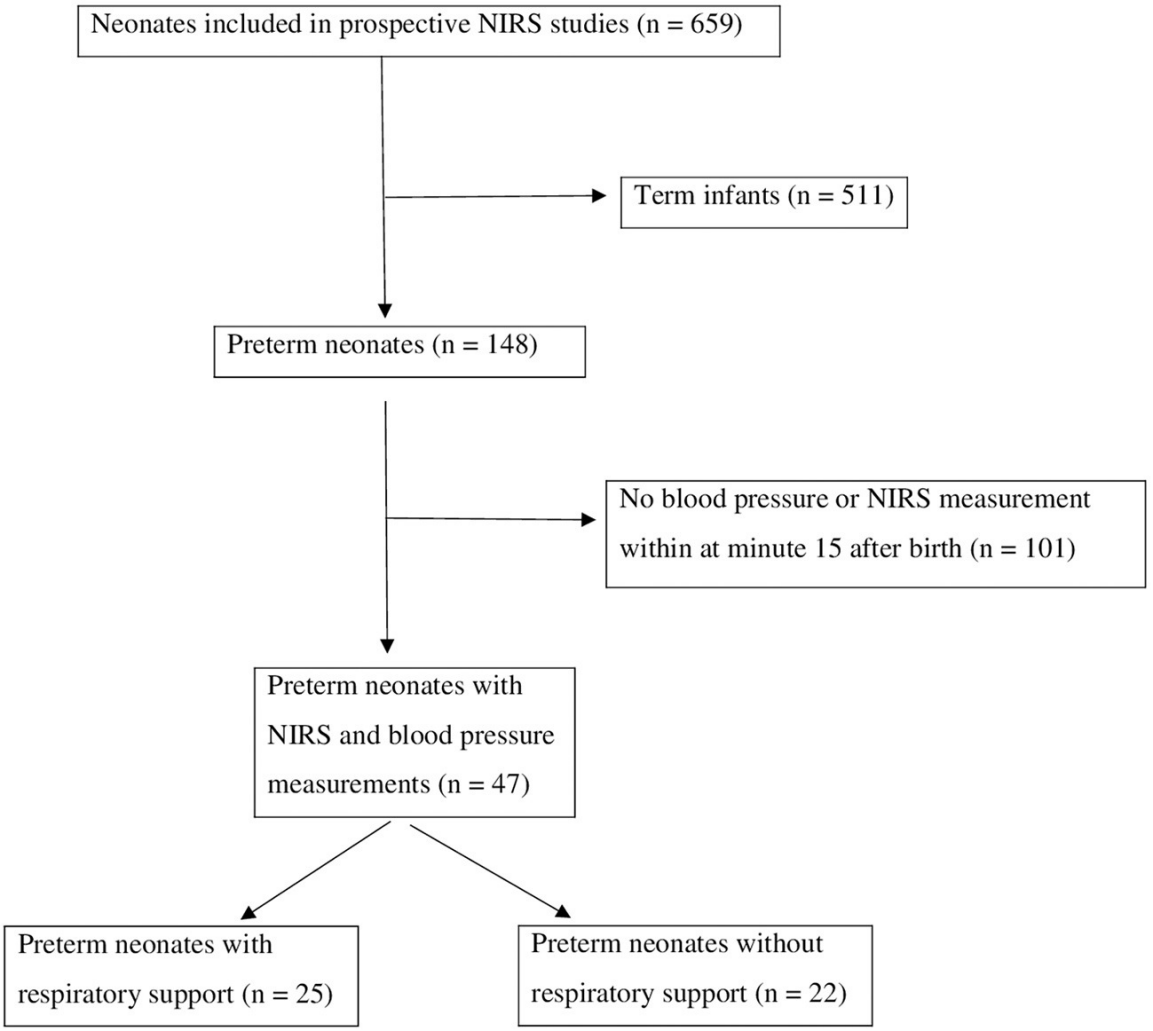
Inclusion and exclusion of eligible neonates.

Twenty-five of these preterm neonates received non-invasive respiratory support and 22 did not receive respiratory support (Figure 1).

None of the neonates received surfactant or cardio-circulatory support (inotropes or volume) during the first 15 min after birth.

Demographic data showed no significant differences between preterm neonates with and without respiratory support except in regard to umbilical artery pH and Apgar scores (Table 1).

**Table 1:** Demographic data.

	**Respiratory support (*n* = 25)**	**No respiratory support (*n* = 22)**	***p*-value**
Gestational age, weeks	34.4 ± 1.6	34.5 ± 1.5	0.828
Birth weight, g	2168 ± 448.7	1988 ± 459.1	0.181
Female sex, *n* (%)	15 (60)	11 (50)	0.491
Umbilical artery pH	7.34 (7.30–7.36)	7.30 (7.28–7.33)	0.019^*^
Apgar 1 min	8 (6–9)	9 (8–9)	<0.001^*^
Apgar 5 min	9 (7–10)	10 (9–10)	<0.001^*^
Apgar 10 min	9 (7–10)	10 (9–10)	<0.001^*^

Preterm neonates with and without respiratory support. Data are presented as mean ± SD, median (IQR), or n (%).

* *p*-values indicate a significant difference between preterm infants with and without respiratory support.

There were no significant differences in the monitoring parameters obtained at 15 min after birth, including SpO2, crSO2, cFTOE, SABP, DABP, and MABP, between the two groups (Table 2). Only HR differed significantly between the two groups, whereby neonates requiring respiratory support had higher HR values compared to those without respiratory support.

**Table 2:** Routine parameters, NIRS parameters and blood pressure parameters of preterm neonates with and without respiratory support.

	**Respiratory support (*n* = 25)**	**No respiratory support (*n* = 22)**	***p*-value**
SpO2 15 min after birth, %	96 (93–98)	96 (93–99)	0.382
HR 15 min after birth, bpm	160 ± 15	151 ± 10	0.025^*^
NIRS parameters			
CrSO2 15 min after birth, %	85 (73–91)	78 (71–84)	0.147
CFTOE 15 min after birth	0.11 (0.07–0.26)	0.18 (0.12–0.26)	0.166
Blood pressure parameters			
SABP 15 min after birth, mm Hg	62 ± 9	61 ± 7	0.706
DABP 15 min after birth, mm Hg	37 ± 10	33 ± 7	0.252
MABP 15 min after birth, mm Hg	46 ± 11	43 ± 6	0.228

Data are presented as mean ± SD, median (IQR), or n (%). SpO2, arterial oxygen saturation; HR, heart rate; NIRS, near-infrared spectroscopy; crS02, cerebral regional tissue oxygen saturation; cFTOE, cerebral fractional tissue oxygen extraction; SABP systolic arterial blood pressure; DABP, diastolic arterial blood pressure; MABP, mean arterial blood pressure.

* *p*-value indicates a significant difference between preterm infants with and without respiratory support.

Table 3 illustrates the correlation analyses of crSO2/cFTOE and SABP/DABP/MABP for preterm neonates with and without respiratory support. Preterm neonates with respiratory support showed significant positive correlations of crSO2 with SABP, DABP and MABP. Furthermore, preterm neonates with respiratory support had significant negative correlations between cFTOE and SABP, DABP and MABP. In contrast, in preterm neonates without respiratory support, neither crSO2 nor cFTOE correlated with the different blood pressure values.

**Table 3:** Correlation analyses of crSO2 and cFTOE with blood pressure 15 min after birth in preterm neonates with and without respiratory support.

	**Respiratory support**				**No respiratory support**			
	**crSO2**		**cFTOE**		**rSO2**		**cFTOE**	
	**r**	**p**	**r**	**p**	**r**	**p**	**r**	**p**
SABP	0.460	0.021	−0.443	0.027	−0.030	0.895	−0.002	0.994
DABP	0.510	0.009	−0.490	0.013	−0.018	0.936	−0.022	0.921

HR did not correlate significantly with crSO2 or cFTOE neither in neonates without respiratory support nor in neonates with respiratory support.

## Discussion

The present study demonstrated a very different pattern of correlation between arterial blood pressure and cerebral oxygenation, whether premature neonates received respiratory support or not. In preterm neonates with respiratory support, higher blood pressure values were associated with higher crSO2 values, whereby cFTOE correlated negatively with blood pressure values. In contrast, these observations were not evident in more stable preterm neonates without respiratory support.

We speculate, that these findings suggest, that passive pressure-dependent cerebral perfusion was present in preterm neonates with need for respiratory support, indicating an impaired cerebral autoregulation in those preterm neonates (24, 25). This would be in accordance with an animal study, which showed impaired autoregulation in preterm sheep during immediate transition (26). Moreover, Baik et al. (20) showed a significant correlation between MABP and cFTOE, but not with crSO2 in human preterm neonates 15 min after birth. The differences in findings, when compared to the present study, may be explained by the fact that Baik et al. did not differentiate, whether preterm neonates received respiratory support or not. Thus, the present study adds the important information that in compromised preterm neonates with need for respiratory support correlations between cerebral oxygenation and blood pressure are more pronounced. The association between cFTOE and HR in neonates with respiratory support is in accordance with the findings concerning blood pressure.

Our findings are also in accordance with observations from the first weeks after birth. In the first five days after birth, Soul et al. (27) demonstrated in their cohort of sick preterm neonates a similar correlation between MABP and cerebral oxygenation due to episodes of impaired autoregulation. Wong et al. (28) presented comparable results regarding MABP and impaired cerebral autoregulation demonstrating that blood pressure variability in preterm neonates within the first 3 days after birth is highly sensitive to cerebral oxygen saturation, resulting from impaired cerebral autoregulation. Hahn et al. (29) described a similar association between arterial hypotension and cerebral oxygenation in preterm neonates within the first 24 h. In addition, Tsuji et al. (30) demonstrated a positive correlation between MABP and cerebral oxygenation in preterm neonates requiring mechanical ventilation in the first 3 days of life. Furthermore, they also showed a strong relationship between impaired cerebral autoregulation and severe intraventricular hemorrhage (IVH) or periventricular leukomalacia. Various study groups confirmed this association and in consideration of neurodevelopmental outcome, the period of impaired cerebral autoregulation correlates with higher short- and long-time mortality and a worse neurodevelopmental outcome (31–34).

Bearing the present findings in mind, we suggest a greater emphasis on blood pressure measurements especially in preterm neonates receiving respiratory support during the immediate fetal-to-neonatal transition. This emphasis was supported by the consideration that arterial hypertension could potentially lead to IVH during this vulnerable transition period, caused by vasodilation due to the increase of CBF, which could possibly result in vessel rupture (35). Whereas, on the other hand, low blood pressure should be avoided because of its associated decrease in CBF, resulting in hypoxia, and lower crS02 causing a higher risk for IVH in preterm neonates (36).

Thus, the question arises when should blood pressure be measured during the immediate transition period and to what extent blood pressure should be treated. A normative study have helped to outline the blood pressure of healthy infants but not necessarily which blood pressure should be targeted (14). At the moment many preterm neonates with arterial hypotension—defined as MABP below gestational age in weeks—are receiving treatment after the immediate transition at the NICU (37). This widely performed approach, however, is still under discussion. Batton et al. (38) demonstrated no better in-hospital outcome achieved by a generous treatment of arterial hypotension in preterm neonates Dempsey et al. (36) demonstrated in the HIP trail no major differences in clinical outcomes regarding survival to 36 weeks of postmenstrual age without severe brain injury, whether hypotension was treated or accepted. Contrary to these findings the EPIPAGE 2 cohort study showed an association with higher survival rates without major morbidity and lower rates of severe cerebral damage when antihypotensive treatment was induced (39).

There are hints that neonates with impaired autoregulation benefit from slightly higher blood pressure values, as shown by Fyfe et al. (40). In contrary to this latter hypothesis Thewissen et al. (41) demonstrated that there was no improvement of cerebral oxygenation by increasing mean arterial blood pressure with dopamin. Furthermore, the findings showed a reduced cerebral autoregulatory capacity in hypotensive preterm neonates compared to non-hypotensive preterm neonates and that the duration of hypotension and cerebral hypoxia measured with NIRS is associated with cerebral hemorrhage or death. Taking this into consideration cerebral oxygenation might be of added value to determine blood pressure treatment in hypotensive preterm neonates, as recommended by Dempsey (42). This approach assessing cerebral oxygenation, as well as blood pressure, might also be relevant for the fetal-to-neonatal transition, taking the present findings in neonates with respiratory support into account. NIRS has been proven to be a feasible tool to monitor continuously and non-invasively cerebral oxygenation and to improve in combination with dedicated interventions the burden of cerebral hypoxia immediately after birth (21, 43).

During fetal-to-neonatal transition, an open ductus arteriosus and its influence on cerebral oxygenation and blood pressure parameters must also be considered. In term infants, the ductus arteriosus generally closes within the first 48 to 72 h after birth (44). Urlesberger et al. (45) observed an effect of the ductus arteriosus during the immediate transition period. Significantly higher crSO2 values within the first 15 min after birth were observed, when a left-to-right shunt *via* ductus arteriosus was present. Differences in ductus arteriosus diameter and shunt behavior in preterm neonates with and without respiratory support 15 min after birth were not ruled out or accounted for in the present study.

Besides that, the respiratory support also needs to be taken in consideration, because it influences the cerebral oxygenation as well as the blood pressure, as shown by Schwaberger et al. (46). Positive end-expiratory pressure (PEEP) leads to a positive intrathoracic pressure. Due to this pressure the venous bloodstream back to the heart is altered and this leads to a decrease in stroke volume. The latter influences the blood pressure as well as the crS02 and may have had influence on the present findings.

## Limitations

There were some limitations to this study. Firstly, the number of analyzed neonates is relatively low, although correlation analyses still demonstrated a distinctly different behavior between the groups. Secondly, cardiac output influencing blood pressure as well as CBF was not measured in the prospective observational studies due the difficulties to perform Doppler sonography during the first 15 min after birth. Therefore, correlation analyses of blood pressure and CBF were not possible to evaluate cerebral autoregulation in more detail. However, in the present study parameters that could be obtained in routine, even during fetal-to-neonatal transition, were analyzed. Thirdly, the influence of the ductus arteriosus and of the respiratory support on arterial blood pressure values immediately after birth, could only be estimated, because no routine echocardiography had been performed in the observational studies.

## Conclusion

This study demonstrated that crSO2 and cFTOE correlated with arterial blood pressure in moderate and late preterm neonates, who received respiratory support during fetal-to-neonatal transition. In contrary there was no such correlation in preterm neonates without respiratory support. These findings are in line with a passive pressure-dependent cerebral perfusion due to impaired autoregulation in compromised neonates with respiratory support.

## Data availability statement

The raw data supporting the conclusions of this article will be made available by the authors, without undue reservation.

## Ethics statement

The studies involving human participants were reviewed and approved by Ethics Committee of the Medical University Graz. Written informed consent to participate in this study was provided by the participants' legal guardian/next of kin.

## Author contributions

DP and GP conceived the research idea, finalized the methods, and analyzed the data. DP wrote the first draft. DP, CW, NH, BS, LM, NB-S, BU, and GP contributed to data collection, interpretation of the results, finalizing the manuscript, and approved the final version of the manuscript.

## Conflict of interest

The authors declare that the research was conducted in the absence of any commercial or financial relationships that could be construed as a potential conflict of interest.

## Publisher's note

All claims expressed in this article are solely those of the authors and do not necessarily represent those of their affiliated organizations, or those of the publisher, the editors and the reviewers. Any product that may be evaluated in this article, or claim that may be made by its manufacturer, is not guaranteed or endorsed by the publisher.
