# Supplementary figures and images for: Correlation between arterial blood pressures and regional cerebral oxygen saturation in preterm neonates during postnatal transition-an observational study
Source: Front Pediatr. 2022 Sep 6;10:952703. doi: 10.3389/fped.2022.952703 (PMC9540233; doi:10.3389/fped.2022.952703)

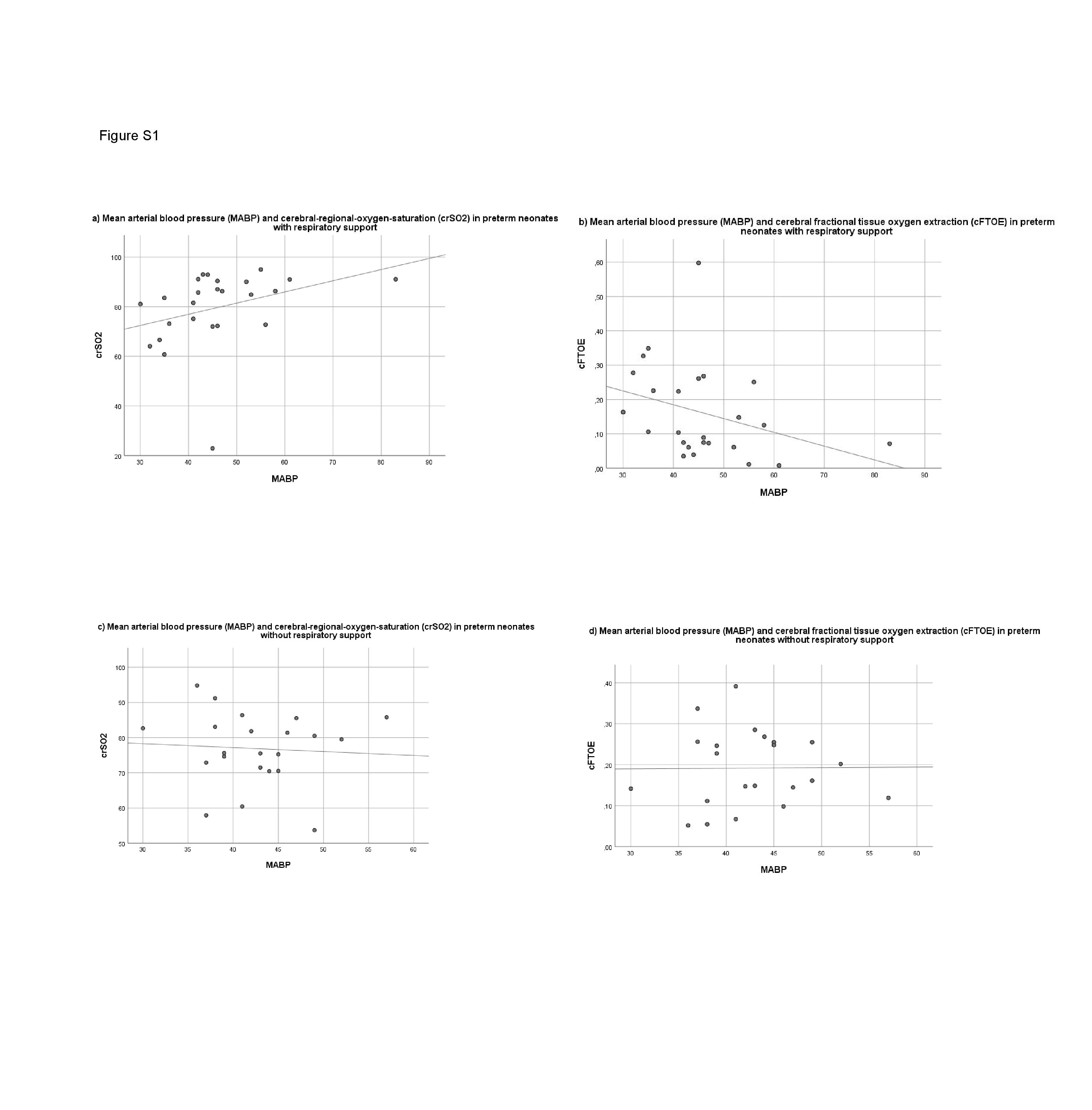

Supplement: Supplementary file 1 [file Image_1.JPEG]

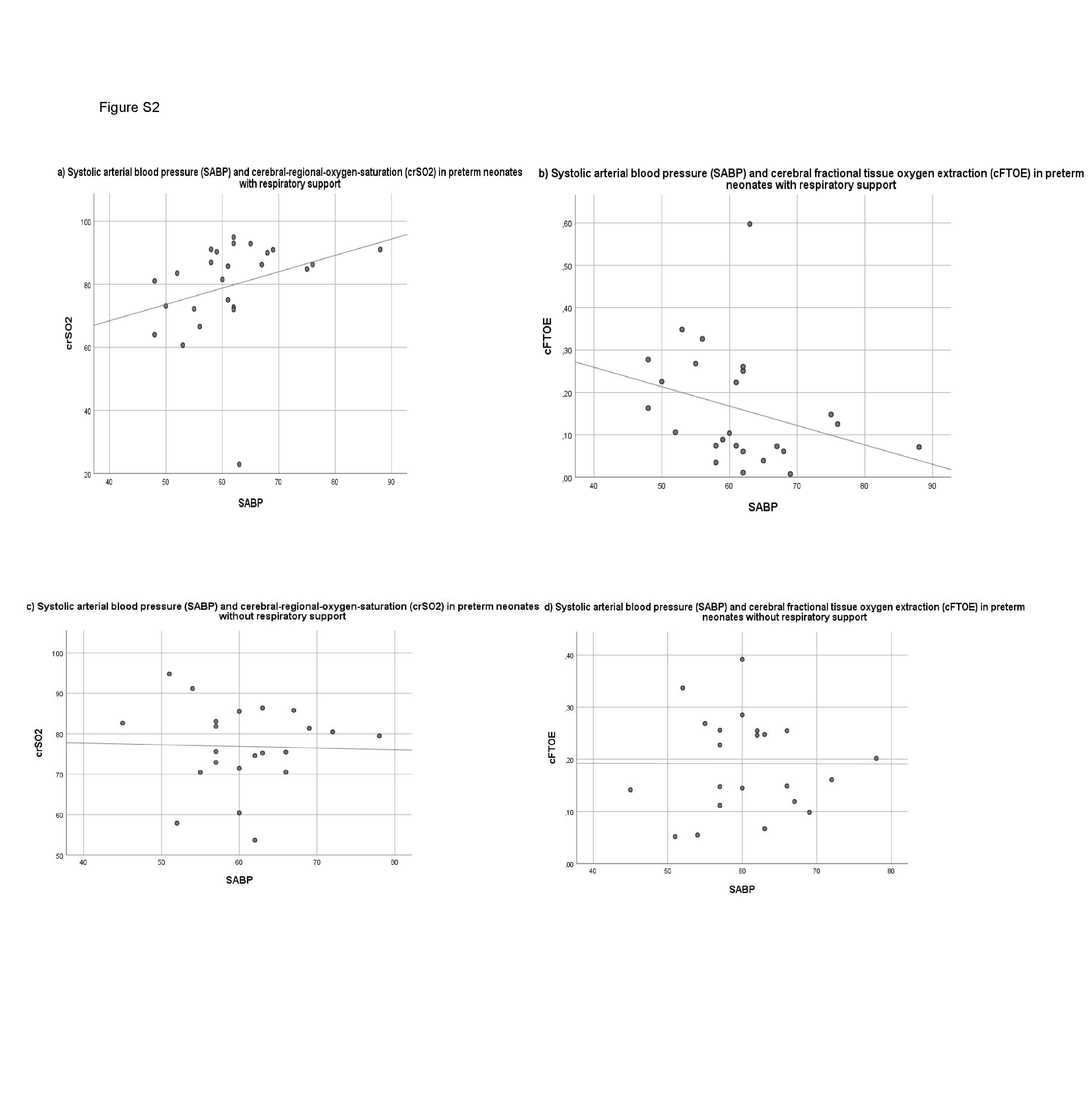

Supplement: Supplementary file 2 [file Image_2.JPEG]

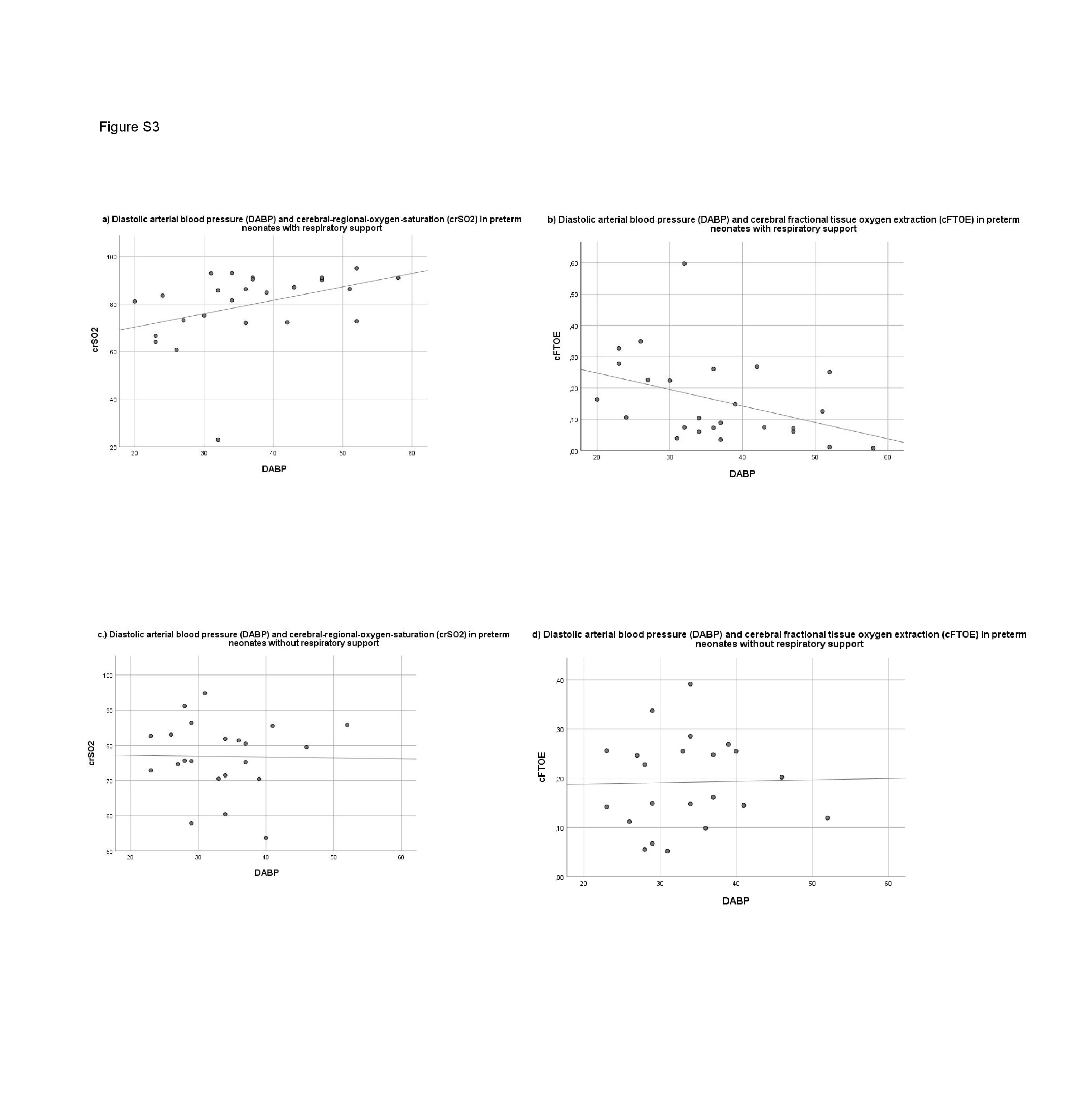

Supplement: Supplementary file 3 [file Image_3.JPEG]
